# Protocol for isolation and characterization of lung tissue resident memory T cells and airway trained innate immunity after intranasal vaccination in mice

**DOI:** 10.1016/j.xpro.2022.101652

**Published:** 2022-08-29

**Authors:** Michael R. D’Agostino, Sam Afkhami, Alisha Kang, Art Marzok, Matthew S. Miller, Zhou Xing

**Affiliations:** 1 McMaster Immunology Research Centre, M.G. DeGroote Institute for Infectious Disease Research, Department of Biochemistry & Biomedical Sciences, McMaster University, Hamilton, ON L8S 4K1, Canada; 2 McMaster Immunology Research Centre, M.G. DeGroote Institute for Infectious Disease Research, Department of Medicine, McMaster University, Hamilton, ON L8S 4K1, Canada

**Keywords:** Cell isolation, Flow Cytometry/Mass Cytometry, Immunology

## Abstract

Vaccination route dictates the quality and localization of immune responses within tissues. Intranasal vaccination seeds tissue-resident adaptive immunity, alongside trained innate responses within the lung/airways, critical for superior protection against SARS-CoV-2. This protocol encompasses intranasal vaccination in mice, step-by-step bronchoalveolar lavage for both cellular and acellular airway components, lung mononuclear cell isolation, and detailed flow cytometric characterization of lung tissue-resident memory T cell responses, and airway macrophage-trained innate immunity.

For complete details on the use and execution of this protocol, please refer to Afkhami et al. (2022).

## Highlights

• Step-by-step procedure for intranasal vaccination in mice
• Protocol for bronchoalveolar lavage and isolating airway cellular/acellular components
• Detailed protocol for processing and isolation of lung mononuclear cells
• Flow cytometry protocol for lung tissue-resident innate/adaptive memory responses

## Abstract (editor highlights)

Publisher’s note: Undertaking any experimental protocol requires adherence to local institutional guidelines for laboratory safety and ethics.

## Graphical abstract

**Figure:**
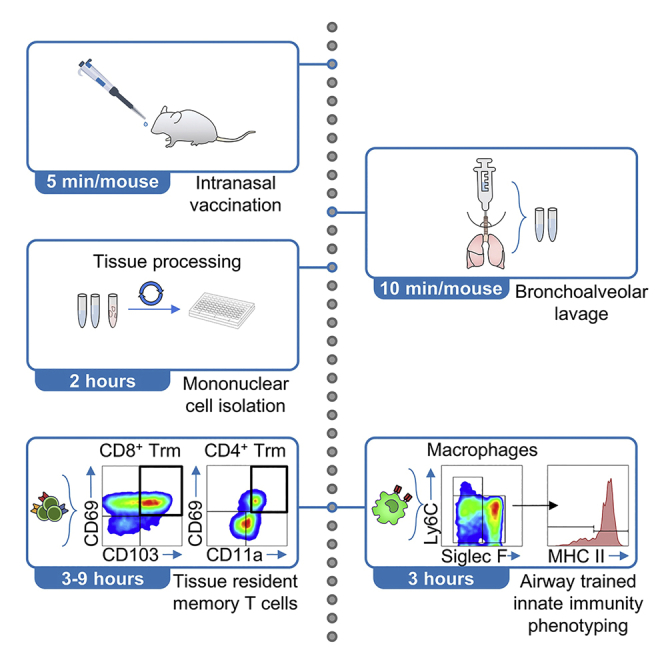

## Before you begin

The experimental techniques and downstream flow cytometric analysis detailed in this protocol are based on samples from mice intranasally vaccinated with a next-generation trivalent chimpanzee adenovirus-vectored COVID-19 vaccine candidate (Afkhami et al., 2022). This protocol has wide applicability and has been utilized to assess immunogenicity from mucosal vaccines for *Mycobacterium tuberculosis* and influenza A virus, primary immune responses to bacterial/viral infection, and to noxious stimuli such as cigarette smoke (Afkhami et al., 2019; D’Agostino et al., 2020; McGrath et al., 2022; Yao et al., 2018). Moreover, widespread use of these standardized techniques will be critical for pre-clinical evaluation of next-generation vaccines or therapeutic treatments for any mucosal pathogen.

### Institutional permissions

All experiments were performed in accordance with institutional guidelines from the Animal Research and Ethics Board.

### Preparation of materials required for bronchoalveolar lavage (BAL)

The following section details how to prepare the necessary materials to perform BAL on a single mouse. Scale accordingly based on experimental need.

**Figure 1:**
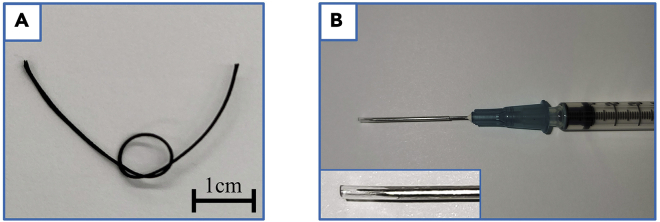
Materials required for bronchoalveolar lavage (A) Suture knot required for bronchoalveolar lavage (BAL). (B) BAL needle design. Note the placement of polyethylene tubing on 23 gauge needle. Magnified figure inset shows the placement of the tubing over the needle bevel.

1. Suture preparation.
  a. Prepare a 4 centimeter (cm) long suture.
  b. Tie an overhand knot with the suture, ensuring at least 1.5 cm on each end (Figure 1A).

**Table 1:** Viability staining

Stain	Antibody/Dye	Stock concentration (mg/mL)	Amount	Temperature	Duration
Viability	Aqua	N/A	1:1000	18°C–25°C	20 min

**Table 2:** Fc Block staining

Stain	Antibody/Dye	Stock concentration (mg/mL)	Amount	Temperature	Duration
Fc Block	Fc Block	0.5	1:100	Ice	15 min

**Table 3:** Trm Master Mix #1 staining

Stain	Antibody/Dye	Stock concentration (mg/mL)	Amount	Temperature	Duration
Trm Master Mix #1	CD103–Biotin	0.5	1:100	Ice	30 min

**Table 4:** Trm Master Mix #2 staining

Stain	Antibody/Dye	Stock concentration (mg/mL)	Amount	Temperature	Duration
Trm Master Mix #2	CD4–APC-Cy7	0.2	1:400	Ice	30 min
	CD8–PE-Cy7	0.2	1:400		
	CD44–PE	0.2	1:800		
	Streptavidin–QDot800	1 μM	1:500		
	CD11a–FITC	0.5	1:100		
	CD49a–AF647	0.2	1:100		
	CD69–BV605	0.2	1:100

**Table 5:** Trm Master Mix #3 staining

Stain	Antibody/Dye	Stock concentration (mg/mL)	Amount	Temperature	Duration
Trm Master Mix #3	CD3–V450	0.2	1:200	Ice	30 min

**Table 6:** Viability staining

Stain	Antibody/Dye	Stock concentration (mg/mL)	Amount	Temperature	Duration
Viability	Aqua	N/A	1:1000	18°C–25°C	20 min

**Table 7:** Fc Block staining

Stain	Antibody/Dye	Stock concentration (mg/mL)	Amount	Temperature	Duration
Fc Block	Fc Block	0.5	1:100	Ice	15 min

**Table 8:** APC Master Mix #1 staining

Stain	Antibody/Dye	Stock concentration (mg/mL)	Amount	Temperature	Duration
APC Master Mix #1	CD64–PE	0.2	1:100	Ice	30 min
	Ly6C–Biotin	0.5	1:200		
	CD11c–APC	0.2	1:200		
	B220–V450	0.2	1:200		
	CD3–PerCp-Cy5.5	0.2	1:200		
	CD45–APC-Cy7	0.2	1:400		
	CD11b–PE-Cy7	0.2	1:400		
	Ly6G–BV605	0.2	1:500		
	CD24–BV650	0.2	1:1500		
	SiglecF–PE-CF594	0.2	1:1500

**Table 9:** APC Master Mix #2 staining

Stain	Antibody/Dye	Stock concentration (mg/mL)	Amount	Temperature	Duration
APC Master Mix #2	Streptavidin–QDot800	1 μM	1:500	Ice	30 min
2. BAL needle preparation.
  a. Prepare 2.5 cm long polyethylene tubing (0.58 mm × 0.965 mm × 30.5 mm).
  b. Prepare a 1 mL syringe equipped with a 23 gauge (G), 1 inch needle.
    i. Using forceps, carefully slide the polyethylene tubing onto the 23G needle and adjust the tubing such that the bevel of the needle is covered, with approximately 0.2 cm of the tubing extending past the tip (Figure 1B).

## Key resources table

**Table:** 

REAGENT or RESOURCE	SOURCE	IDENTIFIER
**Antibodies**		

Purified Rat Anti-Mouse CD16/CD32 (clone 2.4G2) (Fc Block)	BD Biosciences	Cat# 553141, RRID: AB_394656
Anti-mouse CD45 APC-Cy7 (clone 30-F11)	BD Biosciences	Cat# 557659, RRID: AB_396774
Anti-mouse CD11b PE-Cy7 (clone M1/70)	BD Biosciences	Cat# 552850, RRID: AB_394491
Anti-mouse CD11c APC (clone HL3)	BD Biosciences	Cat# 550261, RRID: AB_398460
Anti-mouse MHC II AF700 (clone M5/114.15.2)	Thermo Fisher Scientific	Cat# 56-5321-82, RRID: AB_494009
Anti-mouse CD3 V450 (clone 17A2)	BD Biosciences	Cat# 561389, RRID: AB_10679120
Anti-mouse CD45R (B220) V450 (clone RA3-6B2)	BioLegend	Cat# 103204 RRID: AB_312989
Anti-mouse Ly6C Biotin (clone HK1.4)	BioLegend	Cat# 128004 RRID: AB_1236553
Streptavidin–Qdot800	Thermo Fisher Scientific	Cat# Q10171MP
Anti-mouse CD24 BV650 (clone M1/69)	BD Biosciences	Cat# 563545 RRID: AB_2738271
Anti-mouse CD64 PE (clone X54-5/7.1)	BioLegend	Cat# 139304, RRID: AB_10612740
Anti-mouse Ly6G BV605 (clone 1A8)	BD Biosciences	Cat# 563005 RRID: AB_2737946
Anti-mouse Siglec-F PE-CF594 (clone E50-2440)	BD Biosciences	Cat# 562757, RRID: AB_2687994
Anti-mouse CD4 APC-Cy7 (clone GK1.5)	BD Biosciences	Cat# 552051 RRID: AB_394331
Anti-mouse CD8 PE-Cy7 (clone 53-6.7)	BD Biosciences	Cat# 552877 RRID: AB_394506
Anti-mouse CD44 PE (clone IM7)	BD Biosciences	Cat# 553134 RRID: AB_394649
Anti-mouse CD69 BV605 (clone H1.2F3)	BD Biosciences	Cat# 563290 RRID: AB_2738120
Anti-mouse CD103-Biotin (clone M290)	BD Biosciences	Cat# 557493 RRID: AB_396730
Anti-mouse CD11a FITC (clone 2D7)	BD Biosciences	Cat# 553120 RRID: AB_10892820

**Chemicals, peptides, and recombinant proteins**		

Fetal Bovine Serum (FBS)	Thermo Fisher Scientific	Cat# 16140071
Penicillin-Streptomycin	Thermo Fisher Scientific	Cat# 15140122
L-Glutamine	Thermo Fisher Scientific	Cat# A2916801
Bovine serum albumin (BSA)	Sigma-Aldrich	Cat# 10735086001
RPMI 1640	Thermo Fisher Scientific	Cat# 11875093
MEM Non-Essential Amino Acids Solution	Thermo Fisher Scientific	Cat# 11140050
Sodium pyruvate	Thermo Fisher Scientific	Cat# 11360070
Collagenase type I	Thermo Fisher Scientific	Cat# 17100-017
Red blood cell lysis buffer	Thermo Fisher Scientific	Cat# A10492-01
GolgiPlug (Brefeldin A)	BD Biosciences	Cat# 555029
Aqua Staining Kit	Thermo Fisher Scientific	Cat# L34957
Cytofix/Cytoperm	BD Biosciences	Cat# 554714
HEPES	Thermo Fisher Scientific	Cat# 15630080
Beta-mercaptoethanol	Thermo Fisher Scientific	Cat# 21985023
Trypan blue stain (0.4%)	Thermo Fisher Scientific	Cat# 15250061
Custom peptide pools	Pepscan	N/A

**Software and algorithms**		

FlowJo, version 10	https://www.flowjo.com	SCR_008520

**Other**		

Polyethylene tubing (0.58 mm × 0.965 mm × 30.5 mm)	Becton Dickinson	Cat# 427411
Perma-hand silk surgical suture (size 2-0)	Ethicon Inc	Cat# LA55G
PrecisionGlide 23G (0.6 mm × 25 mm)	BD Biosciences	Cat# 305145
1 mL TB Syringe (Slip tip)	BD Biosciences	Cat# 309659
5 mL Syringe	BD Biosciences	Cat# 309646
Tissue culture plate 96 well, U-bottom	Thermo Fisher Scientific	Cat# 353077
Cell strainer 100 μm Nylon	Thermo Fisher Scientific	Cat# 352360

## Materials and equipment

**Table:** Perfusion solution (optional)

Reagent	Final concentration	Amount
RPMI-1640	N/A	88 mL
FBS (optional)	10%	10 mL
HEPES (optional)	10 mM	1 mL
Penicillin/Streptomycin	100 U/mL Penicillin 100 μg/mL Streptomycin	1 mL
Total	N/A	100 mL

***Note:*** 0.2 μm filter sterilize. Store at 4°C for up to six months. Prepare as per experimental need. Each lung sample can be sufficiently perfused with 5–10 mL of perfusion solution.

**Table:** Lung digestion solution

Reagent	Final concentration	Amount
RPMI-1640	N/A	88 mL
FBS (optional)	10%	10 mL
HEPES (optional)	10 mM	1 mL
Penicillin/Streptomycin	100 U/mL Penicillin 100 μg/mL Streptomycin	1 mL
Collagenase type 1	150 U/mL	Dependent on stock concentration
Total	N/A	100 mL

***Note:*** Ensure the collagenase is completely dissolved. 0.2 μm filter sterilize. Store at 4°C and prepare the day of the experiment. Prepare as per experimental need. Each lung sample requires 10 mL of lung digestion solution.

**Table:** Cell culture media

Reagent	Final concentration	Amount
RPMI-1640	N/A	84.5 mL
FBS	10%	10 mL
HEPES	10 mM	1 mL
Penicillin/Streptomycin	100 U/mL Penicillin 100 μg/mL Streptomycin	1 mL
L-Glutamine	2 mM	1 mL
Non-essential amino acids	0.1 mM	1 mL
Sodium pyruvate	0.1 mM	1 mL
Beta-mercaptoethanol	55 μM	500 μL
Total	N/A	100 mL

***Note:*** 0.2 μm filter sterilize. Store at 4°C for up to 2 weeks. Prepare as per experimental need.

**Table:** FACS buffer

Reagent	Final concentration	Amount
Phosphate buffered saline (PBS)	N/A	100 mL
BSA	0.5%	0.5g
Total	N/A	100 mL

***Note:*** 0.2 μm filter sterilize. Store at 4°C for up to one month.

## Step-by-step method details

### Experimental procedure #1: Intranasal vaccination

This section details the handling, preparation, and intranasal delivery of liquid agents in the murine model (see Methods video S1). Although this protocol utilizes adenoviral-vectored vaccines as the model system for demonstration purposes, this procedure applies to delivery of any liquid solution such as viruses, bacteria, antibodies, drugs, etc.

**Timing: 5 min per mouse**

**Figure 2:**
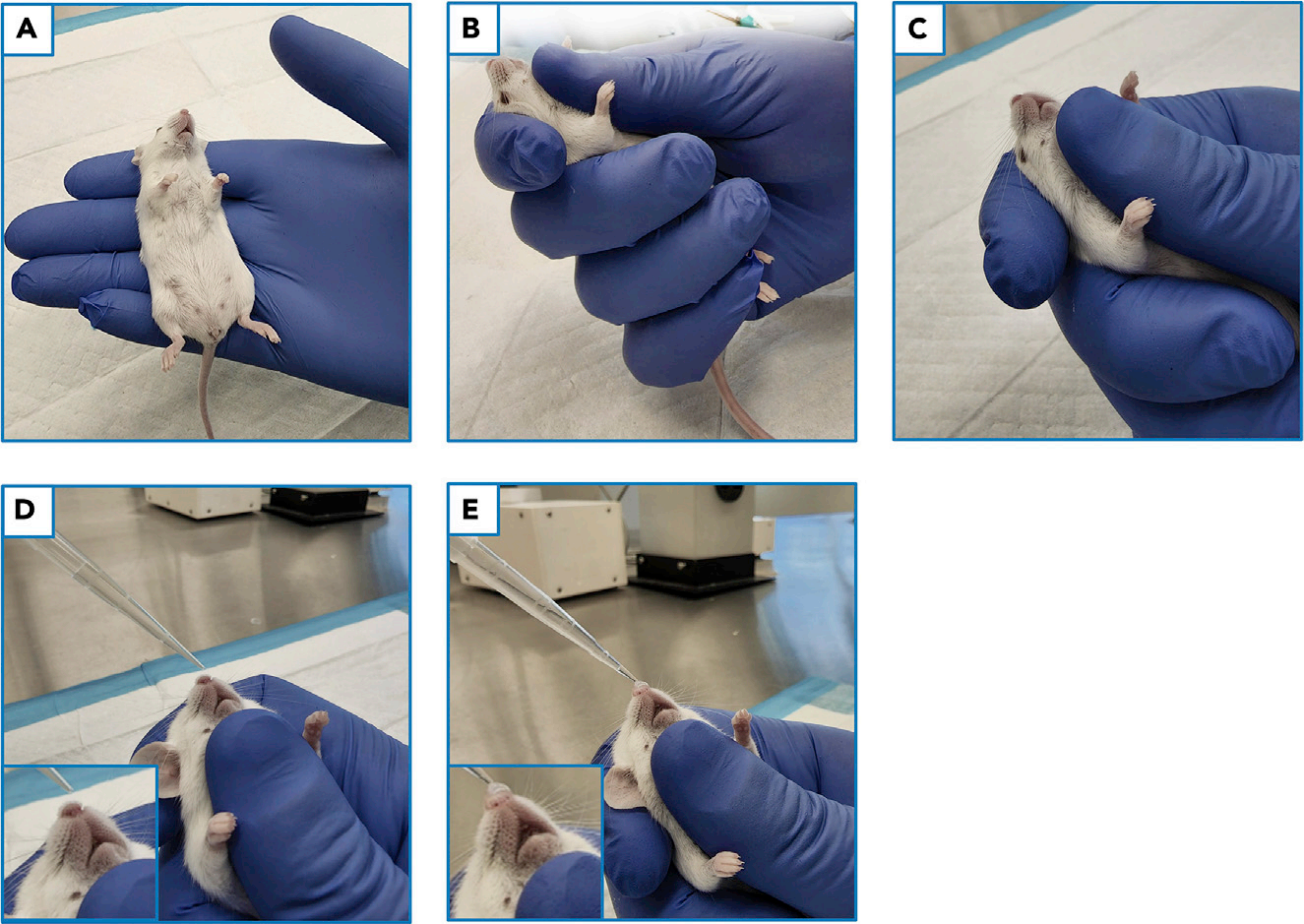
Mouse handling and intranasal vaccination procedure (A) Mouse placement in the palm. (B) Thumb placement on the jaw and throat. (C) Mouse positioning and thumb placement on the jaw and throat. (D) Pipette tip positioning over the nare prior to intranasal vaccination. Note thumb positioning. (E) Droplet on the nose during intranasal vaccination. Note thumb positioning.
  b. Intranasal instillation should be performed when the mouse breathes once every 2–3 s.

***Note:*** When using gaseous anesthesia, the oxygen and anesthetic flow rates directly affect the respiration rate of mice in the anesthesia chamber. Researchers should first familiarize themselves with their specific equipment.

1. Anesthetize mice with an approved inhaled or injectable anesthetic (see note).
  a. Once mice are anesthetized, retrieve one from the anesthetic chamber and place the mouse in your hand to observe its respiration rate (Figure 2A).

**CRITICAL:** Regulation of the mouse’s breathing rate is imperative to ensure optimal timing for intranasal instillation. Gaseous anesthesia allows for more refined control of breathing rate and is recommended over injectable anesthesia.

2. Once anesthesia is achieved, load the pipette with the agent set to be intranasally administered and set it aside until step 7.

***Note:*** Depending on the volume of the liquid to be instilled, a single bolus of 25 μL, or two 20 μL boluses may be delivered. Additionally, the volume of liquid administered affects distribution in the lung. The larger the volume, the deeper in the lung it will reach, but the more difficult it will be to instill the entire volume (Southam et al., 2002).

3. Place and position the mouse on its back in the palm of your non-dominant hand (Figure 2A).
4. Using your thumb, trace along the midline of the mouse, over the xyphoid process/sternum, up the throat, and onto the jaw and throat (Figure 2B).
  a. Apply light pressure.

**CRITICAL:** Pay close attention to how the mouse is placed in your non-dominant hand (Figure 2A). Additionally, it is imperative that the jaw remains tightly closed to prevent the mouse from swallowing the intranasally administered liquid. Researchers should first practice and familiarize themselves with this process. In general, observation of the mouse’s respiration without visible swallowing is an indicator of proper thumb positioning and pressure.

5. Angle the supine-positioned mouse at approximately a 60° incline, ensuring your thumb remains depressed on the jaw (Figure 2C).
6. Holding the mouse in this position, re-examine its breathing rate.
  a. If the mouse is not breathing at this point, adjust your thumb positioning and re-apply pressure, ensuring that rhythmic breathing is maintained.
7. Once a rhythmic breathing rate (as described in step 1a) is observed, take the loaded pipette with your dominant hand, and position the pipette tip just above the nares (Figure 2D).
8. Depress the pipette plunger to slowly instill a droplet onto the mouse’s nares (Figure 2E).
  a. Wait for the mouse to take a breath, inhaling the droplet.
  b. Once the droplet has been inhaled, wait for an additional breath before proceeding with the next droplet to ensure the first droplet is fully internalized.
9. Once the droplet has been inhaled, maintain the supine mouse in the 60° position for another 2–3 breaths to ensure the inhaled liquid does not bubble back out of the nose.
  a. If bubbling is observed, the liquid can be aspirated by pipette and re-administered, as described in steps 7 and 8.
10. Remove your thumb from the mouse’s jaw and place it on the mouse’s chest.
11. Depress lightly. If the droplet has been efficiently inhaled, turbulence should be felt with every breath.
12. Return the mouse to its cage and place it on its back to recover.

Methods video S1. Intranasal instillation of a liquid bolus into an anesthetized mouse, related to Experimental procedure #1, steps 3–1 1

### Experimental procedure #2: Bronchoalveolar lavage

This section details how to excise and prepare the lungs for extrathoracic BAL, and secondly describes how to process the BAL fluid for isolation and downstream utilization of acellular and cellular airway components.

**Timing: 10 min per mouse**

13. Mouse preparation, lung excision, and BAL needle placement.
  a. Anesthetize mice and maintain anesthesia as per animal research ethics board-approved protocols.
    i. Either gaseous or injectable anesthesia may be used.

**CRITICAL:** Avoid anesthetizing multiple mice at once. Do not sacrifice the mouse by cervical dislocation as this may damage the trachea and affect the integrity of the BAL procedure.

**Figure 3:**
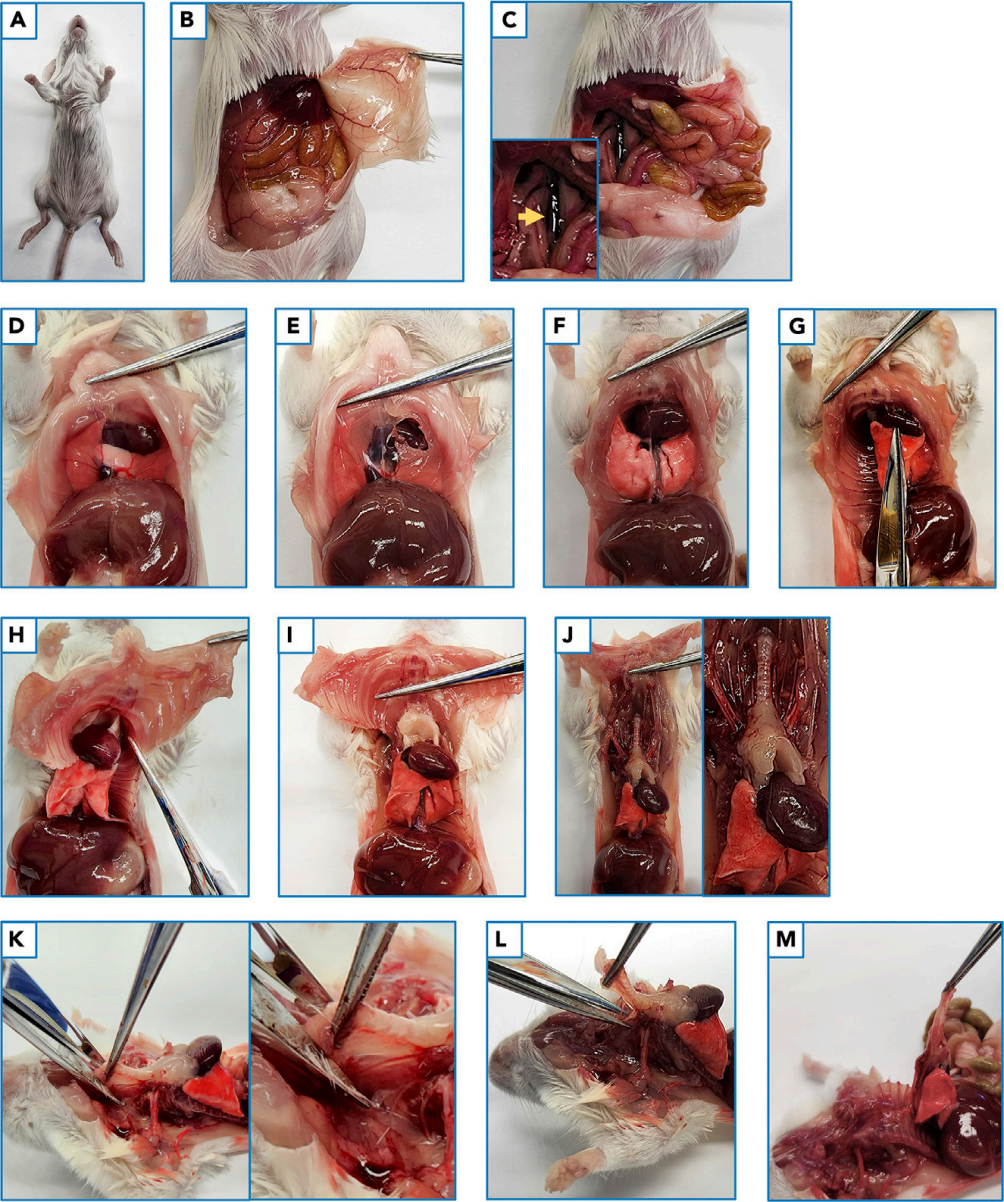
Exsanguination, lung extraction and preparation for bronchoalveolar lavage (A) Mouse orientation and surgical site preparation with 70% ethanol. (B) Abdominal incision. (C) Abdominal organ displacement and artery visualization. Magnified inset of the artery (yellow arrow). (D) Visualization of the intact thoracic diaphragm, with the liver displaced from it. (E) Perforation of the thoracic diaphragm. Visualization of lung collapse. (F) Dissection of the thoracic diaphragm. (G) Displacement of the lungs from the rib cage. (H) Dissection of the rib cage. (I) Forceps placement on dissected rib cage. (J) Visualization of the intact trachea. (K) Scissor and forceps placement for tracheal dissection. (L) Connective tissue dissection for removal of the thoracic block. (M) Removal of the thoracic block.
16. Using forceps and surgical scissors, make an incision into the abdomen, continuing towards the rib cage (Figure 3B).
17. Using forceps, laterally displace the abdominal organs, exposing the abdominal artery (Figure 3C).
18. Euthanize the mouse by sharply dissecting the abdominal artery.
  a. Absorb the blood using sterile gauze, or alternatively, blood may be collected directly from the artery using a 27G needle and syringe.
19. Using surgical scissors, dissect the liver from the diaphragm (Figure 3D).
20. Carefully perforate the thoracic diaphragm without touching the lungs.
  a. At this point the lungs should collapse, retracting away from the diaphragm (Figure 3E).
21. Completely dissect the diaphragm (Figure 3F).
22. Using either forceps or the flat side of surgical scissors, gently push the lungs medially, separating them from the rib cage (Figure 3G).
23. Using surgical scissors, dissect superiorly through both lateral aspects of the rib cage towards the clavicles (Figure 3H).
24. Using forceps, grip the ribcage (Figure 3I).
25. With one hand firmly grasping the base of the mouse’s tail, pull the ribcage superiorly towards the head to expose the intact trachea (Figure 3J).
  a. Use sterile gauze to clean the exposed trachea if any residual bleeding is observed. This clean up step is essential to minimize contamination of the BAL fluid with blood.

***Note:*** If the trachea remains unexposed after step 25, sharply dissect the tissues in the throat region to expose the intact trachea. Alternatively, in place of step 25, one may instead sharply dissect away the rib cage and throat tissue to reveal the intact trachea. However, this latter procedure is more time consuming, and you risk accidentally cutting the trachea.

14. Once the mouse is anesthetized, place it in a supine position.
15. Soak the abdominal and thoracic regions with 70% ethanol to disinfect the area and prevent fur from attaching to surgical equipment (Figure 3A).

26. Using forceps, grip the most superior part of the trachea, immediately inferior to the larynx (Figure 3K).
27. Sharply dissect the trachea with surgical scissors superior to where the forceps are grasping the trachea (Figure 3K).
28. While holding the trachea with forceps, gently pull the trachea distally while cutting away any connective tissue that is revealed along the ribcage (Figure 3L).
  a. Continue this process until the entire thoracic block is freed from the connective tissue and can be lifted cleanly out of the mouse (Figure 3M).

**CRITICAL:** Proceed slowly. Pulling too hard or too quickly risks tearing the trachea or lung tissue which can compromise BAL recovery.

**Figure 4:**
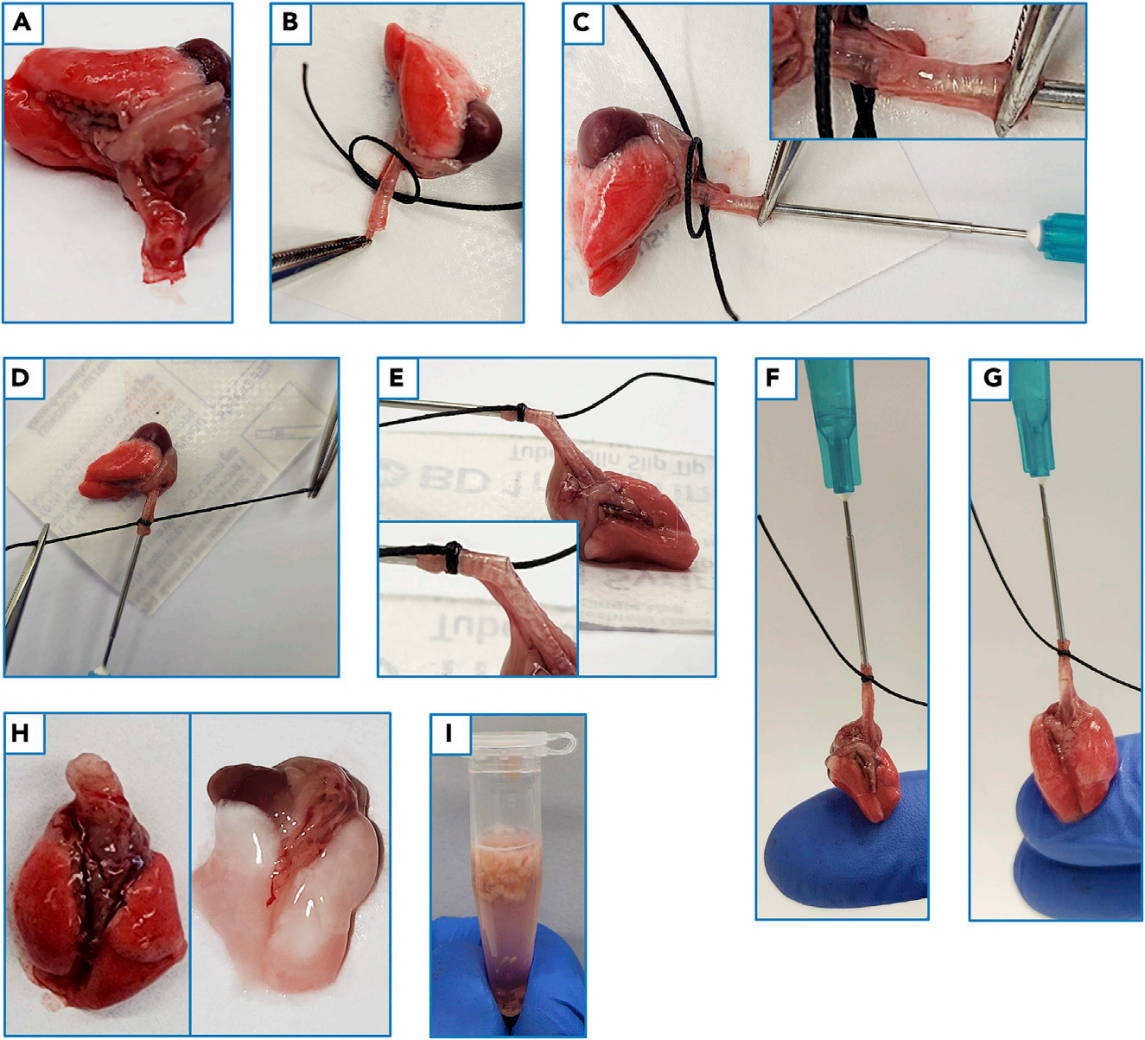
Lung cannulation, suturing, and bronchoalveolar lavage (A) Lung and tracheal placement. (B) Suture positioning over the trachea. (C) Cannulation of trachea with the BAL needle. Inset showing depth of BAL needle in the trachea. (D) Tightening of suture, securing the trachea to the BAL needle. (E) Visualization of BAL needle positioning in the trachea. (F) Upright positioning and lung support prior to BAL. (G) Lung inflation during BAL. (H) Lungs prior to, and after perfusion. (I) Lung tissue after mincing in 1.5mL collection tube.
30. Using forceps, pass the trachea through the loop of the prepared surgical suture (Figure 4B).
31. Load a BAL needle with 250 μL of sterile, ice-cold PBS.
32. While guiding the trachea with the forceps, insert the BAL needle into the opening of the trachea (Figure 4C).

***Note:*** Avoid the esophagus when cannulating. The trachea is distinguishable by the presence of cartilaginous rings. The needle should be positioned approximately halfway down the length of the trachea. The location of the needle can be visualized by lifting the needle up to determine the depth in the trachea (Figure 4C).

29. Place the thoracic block onto a piece of wax paper (e.g., from the packaging of a single-wrapped 1 mL syringe) so the trachea can be laid straight and flat along the paper (Figure 4A).

**CRITICAL:** Ensure the sharp, beveled end of the needle is entirely concealed by the polyethylene tubing to avoid puncturing the trachea.

33. Tighten the surgical suture around the trachea, where it overlaps with the metal shaft of the needle (Figure 4D).
34. Once the suture is tightened, securing the trachea to the BAL needle, check the depth of the needle in the trachea, and the alignment of the surgical suture such that it sits just below the opening of the trachea and on the metal shaft of the BAL needle (Figure 4E).
  a. Forceps can aid in this process by holding the tracheal tissue in place.

**CRITICAL:** Tie the suture as tight as possible to ensure the trachea does not slip off the needle during the BAL procedure.

35. Conventional bronchoalveolar lavage.
  a. Once the suture has been securely tightened around the trachea and BAL needle, lift the lungs off the wax paper with the syringe. Support the lungs so they are not freely hanging by placing a finger underneath the lungs (Figure 4F).
  b. Hold the syringe in a vertical position such that the trachea is aligned with the needle (avoiding any visible kinks) (Figure 4F).
36. Gently depress the plunger of the syringe. The lung lobes should begin to inflate on both sides.
  a. The angle of the BAL needle may need to be adjusted to ensure that both the left and right lobes inflate (Figure 4G).
37. Gently pull back on the syringe plunger to aspirate the instilled PBS. The lungs may be gently massaged while pulling back on the plunger to aid in lavage fluid retrieval.

***Note:*** Approximately 100–200 μL of the first 250 μL instillation of PBS is retrievable. If resistance is felt when pulling the plunger back, the positioning of the BAL needle may need to be adjusted. If so, place the lungs back down on the wax paper and gently readjust the BAL needle positioning. This may entail moving the needle back and forth to work around or through the blockage. Repeat steps 35–37.

38. Detach the syringe from the BAL needle and eject the recovered volume into a 1.5 mL collection tube.
39. With the same syringe, aspirate an additional 200 μL of ice-cold PBS and reattach it to the BAL needle.
40. Repeat steps 35–37 and eject the recovered volume into the same the 1.5 mL collection tube as used in step 38. Place the collection tube on ice.

***Note:*** A total recovery volume of 300–400 μL is expected after the first two instillations of PBS.

41. Exhaustive bronchoalveolar lavage.
  a. With the same syringe that was used for conventional BAL in steps 35–37, aspirate 300 μL of ice-cold PBS and reattach it to the BAL needle.
42. Repeat the BAL procedure as per steps 35–37 and eject the recovered volume into a fresh 1.5 mL collection tube.
43. Repeat this BAL procedure with two additional instillations of 300 μL of ice-cold PBS each time. Eject the recovered volume into the same 1.5 mL collection tube as step 42 (900 μL total volume) and place the tube on ice.
44. Following BAL, dissect the lung lobes with scissors and forceps and place the pieces into pre-labeled 1.5 mL lung collection tubes filled with 750 μL cell culture media and place the tube on ice.

***Optional:*** Prior to dissection of the lung lobes from the thoracic block, perfusion may be performed. Perfusion is performed by injecting 5 mL of ice-cold perfusion buffer into the right ventricle of the heart with a 23G needle. Successful perfusion should visibly blanch the lung tissue of red blood cells (RBC), with the tissue taking on a predominately white/translucent appearance (Figure 4H).

***Note:*** Perfusion does not remove vascular-bound leukocytes. To properly differentiate vascular-restricted vs. bona fide parenchymal leukocytes, intravenous labelling is required, as described in (Anderson et al., 2012, 2014).

45. Incubate samples on ice until all samples have been collected to maximize cell viability.
46. Processing of BAL fluid for downstream applications.

***Note:*** The above protocol separates the BAL fluid into conventional and exhaustive fractions. The conventional fragment is enriched in both soluble and cellular components, while the exhaustive fraction is primarily cellular components.

47. Centrifuge the 1.5 mL collection tubes containing the conventional and exhaustive BAL fluid at 3,000 × *g*, 4°C, for 5 min in a tabletop microcentrifuge.
48. After centrifugation, visualize the cell pellets. Transfer the supernatant from the conventional BAL fraction to a fresh 1.5 mL collection tube and store samples at −20°C.
49. Decant the supernatant from exhaustive BAL fraction into liquid waste with a pipette.
50. Resuspend the cell pellet from the conventional BAL fraction in 200 μL of cell culture media.
  a. Combine this cell suspension with the cell pellet from the exhaustive BAL fraction and resuspend. Store samples on ice until required for downstream applications.
51. Enumerate cells and determine viability by trypan blue staining.
  a. On average, 3–5 × 10^5^ cells are expected to be recovered from a naïve mouse.

### Experimental procedure #3: Lung mononuclear cell isolation

This section details how to process lung tissues for isolation of mononuclear cells for downstream flow cytometric analysis. Cells isolated by this method should retain greater than 95% viability and are suitable for other biological assays such as RNA isolation, *ex vivo* culture, etc.

**Timing: 2 h**

52. Once all lung samples have been collected, mince the lung tissue into smaller pieces using surgical scissors in the 1.5 mL lung collection tube (Figure 4I).

***Note:*** A standard drill-bit or bead-based tissue homogenizer will reduce sample viability; as such, tissue processing must be carried out manually.

53. Transfer the minced tissue into a sterile 50 mL polypropylene tube.
54. Using a serological pipette, dispense 10 mL of lung digestion solution into the 50 mL polypropylene tube containing the minced lung tissue.
  a. Minced tissue that remains in the 1.5 mL tube can be rinsed out with lung digestion solution.
55. Incubate samples in a 37°C shaking incubator at 225 rpm for 1 h.

***Note:*** If a shaking incubator is unavailable, the samples may be incubated at 37°C and manually swirled every 10–15 min for the duration of the incubation. Researchers must swirl carefully to minimize the amount of lung tissue that clings to the side of the polypropylene tube, which can prevent it from being fully submerged in lung digestion solution.

56. Following incubation, pour samples through a 100 μm nylon mesh basket filter into a fresh sterile 50 mL polypropylene tube.
57. Grind the digested tissue through the basket filter with the rubber end of a plunger from a 5 mL syringe.
  a. As the tissue is ground through the filter, occasionally rinse the filter with ice-cold RPMI-1640 to wash cells through the filter.
58. Repeat step 57 until all visible pieces of lung tissue have been ground through the filter.

***Note:*** Some fibrous tissue is expected to remain in the filter after this process. Researchers should use a separate filter for each sample to avoid clogging the filter.

59. Centrifuge samples at 500 × *g* at 4°C for 5 min.
60. After centrifugation, visualize the cell pellet, then decant the supernatant in one motion into a liquid waste container.
61. Resuspend the cell pellet with 1 mL of RBC lysis solution. Once fully resuspended, add an additional 1 mL of RBC lysis solution to the sample.
  a. Incubate samples for 2 min at 18°C–25°C. Occasionally agitate samples by gently swirling the tubes.

***Note:*** Researchers should work quickly during RBC lysis. If multiple samples are being handled, dividing the workflow will ensure greater uniformity in lysis duration between samples.

62. Stop the lysis reaction by adding 40 mL of ice-cold PBS to each sample.
63. Filter samples through a 100 μm nylon mesh basket filter into a new sterile 50 mL polypropylene tube. White/translucent fibrous tissue will be present in the filter.
64. Centrifuge samples at 500 × *g* at 4°C for 5 min.
65. After centrifugation, visualize the cell pellet, then decant the supernatant in one motion into a liquid waste container.
  a. Leave the tube momentarily inverted on a stack of low-particulate wipes to remove additional supernatant.
66. Resuspend cells in 1 mL of complete cell culture media. Place samples on ice.
67. Enumerate cells and determine viability by trypan blue staining.
68. For downstream flow cytometric analysis, resuspend cells to a final concentration of 2 × 10^7^ cells/mL. Cells are now ready for downstream utilization.

### Immunological procedure #1: Mononuclear cell phenotyping of tissue resident memory T cell responses

**Figure 5:**
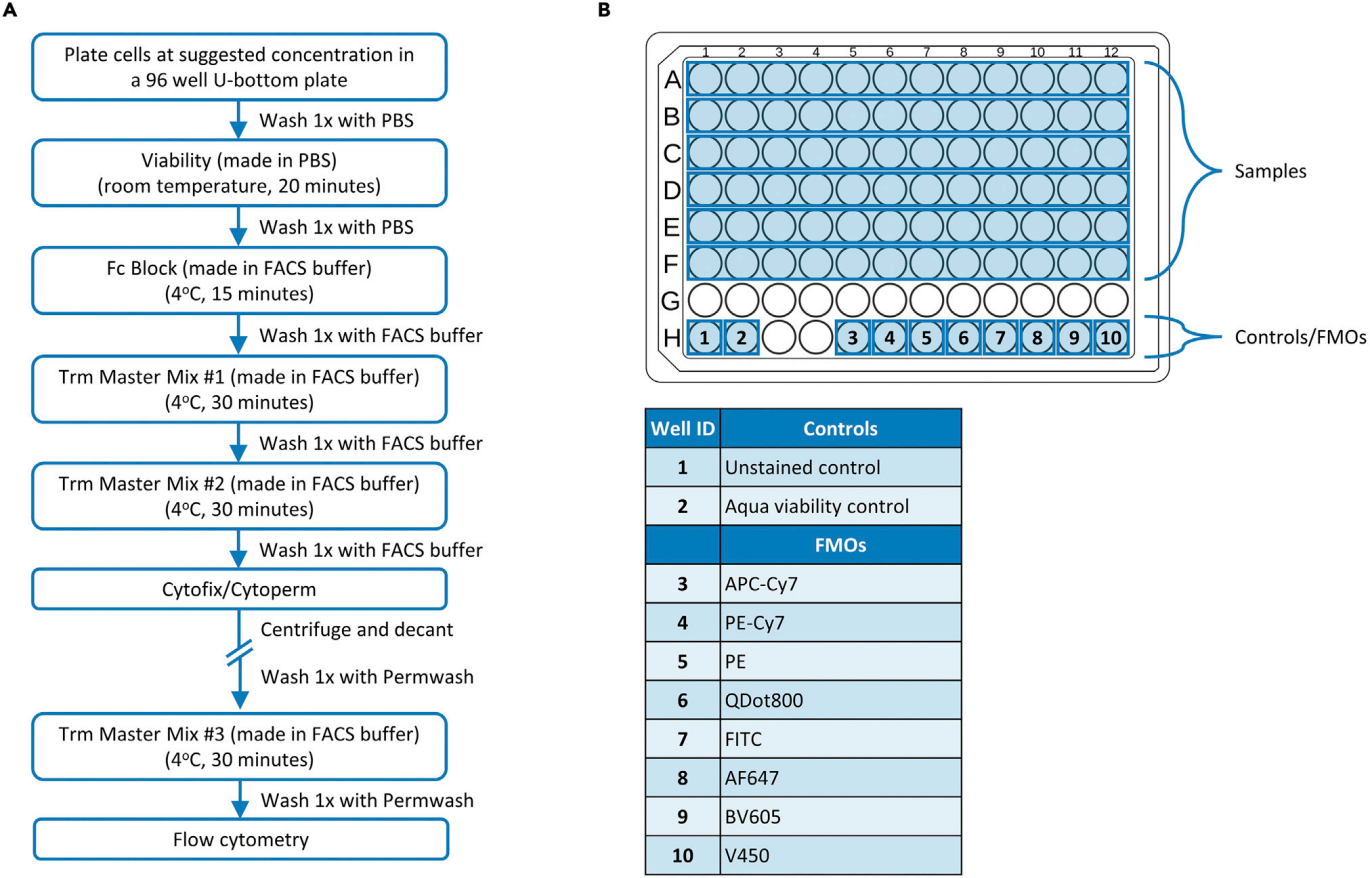
Staining strategy for identification of tissue resident memory T cell responses (A) Staining workflow. (B) Example plate layout.

This section details the staining of BAL and lung mononuclear cells for identification of antigen-specific tissue resident memory (Trm) T cell responses by flow cytometry. The antibody cocktail described herein is a suggestion for canonical Trm markers using specific antibodies that we have experimentally proven to work. Researchers may need to adjust the panel as per experimental need. The staining workflow for Trm analysis is presented in Figure 5A.

**Timing: 3 h without stimulation, 9 h with stimulation**

69. Mononuclear cell plating.
  a. Add volume/concentration-adjusted BAL and lung mononuclear cells to a 96 well U-bottom plate. To ensure enough events can be collected on the flow cytometer for sufficient data resolution, researchers are encouraged to: (1) Plate 5 × 10^5^–2 × 10^6^ lung mononuclear cells, and (2) 3–5 × 10^5^ BAL cells. Volume-adjust cells to 200 μL.
    ***Note:*** Lung mononuclear cells may be utilized for Fluorescence Minus One (FMO)/isotype controls. Researchers are encouraged to prepare these for each color to ensure proper quality control of positive population cut offs (Figure 5B). Unstained and color controls made from lung mononuclear cells can also be used for compensation of BAL samples.
    ***Optional:*** Peptide stimulation preparation.
    ***Note:*** Instead of including broad activation markers such as CD44 in the flow cytometry panel, researchers may choose to directly assess antigen-specific T cell responses. This can be achieved by stimulating mononuclear cells with peptide pools spanning the antigen-of-interest and subsequently probing for cytokine production such as interferon gamma (IFNγ).
    Preparation of peptide stimulants for assessing antigen-specific T cell responses is dependent on the researcher’s needs. In this protocol subsection, overlapping 15mer peptide libraries with 10 amino acid overlaps were commercially generated at a purity of greater than 70%. This approach allows for quantification of both CD4^+^ and CD8^+^ antigen-specific T cell responses and can be utilized for downstream epitope mapping studies.
    Stimulations are performed using peptide pools containing overlapping peptides spanning the antigen-of-interest. Stimulations are performed at a final concentration of 2 μg/mL for each peptide. In this protocol, stimulations are performed in 96 well U-bottom plates with a final stimulation volume of 200 μL.
    The following procedure should be carried out immediately after cell enumeration, and prior to any flow cytometry staining.
    [peptide pool stock]×(volume of peptide pool stock) = 2 μg/mL × 0.2 mL.
  b. Prepare stimulants at a final volume of 50 μL per condition with cell culture media.
  c. Add volume/concentration-adjusted lung/BAL mononuclear cells to the 96 well U-bottom plate and mix with the stimulant.
  d. Prepare a working solution of Brefeldin A as per manufacturer’s instructions such that a final volume of 50 μL is added per well with cell culture media.
  e. Add the Brefeldin A working solution to the cell and stimulus mixture. Ensure the final volume of each well is 200 μL by adding more cell culture media as needed.
  f. Incubate the plate at 37°C, 5% CO_2_ for 6 h.
    Following incubation, proceed to step 70.
    ***Note:*** Ensure that the CD44 antibody is replaced with a color-compatible cytokine antibody detection cocktail. Gate on the cytokine positive population prior to assessing Trm markers.
70. Viability LIVE/DEAD staining protocol.
  a. Centrifuge the plated cells at 500 × *g* at 4°C for 5 min.
  b. After centrifugation, visualize cell pellets then decant the supernatant by flicking the plate in one singular motion into a liquid waste container.
    i. Leave the plate momentarily inverted on a stack of low-particulate wipes to remove additional supernatant.
  c. Resuspend cell pellets with 200 μL of ice-cold PBS and centrifuge the plate at 500 × *g* at 4°C for 5 min.
    ***Note:*** The presence of serum/protein in the cell culture media may impact the resolution of amine reactive viability dyes. Researchers are encouraged to perform at least a single wash step with PBS prior to staining with amine reactive viability dyes as per manufacturer’s instructions.
  d. Prepare the viability stain working dilution in PBS as per manufacturer’s instructions. Add 50 μL of the viability stain working solution to each well containing samples, except the unstained control (Table 1).
  e. Incubate the samples at 18°C–25°C for 20 min, protected from light.
    ***Note:*** For compensation, researchers may either prepare a cell color control stained with the viability dye or may utilize amine reactive beads (e.g., ArC amine reactive beads: Thermo Fisher Scientific cat# A10346).
  g. Following incubation, add 150 μL of ice-cold PBS to each well and centrifuge the plate at 500 × *g* at 4°C for 5 min.
  h. After centrifugation, visualize cell pellets, then decant the supernatant by flicking the plate in one motion into a liquid waste container.
  i. Leave the plate momentarily inverted on a stack of low-particulate wipes to remove additional supernatant.
71. Fc Block staining protocol.
  a. Prepare the working dilution of Fc block and resuspend samples in 50 μL of the working solution (Table 2).
  b. Incubate samples for 15 min on ice, protected from light.
  c. Following incubation, add 150 μL of ice-cold FACS buffer to each well and centrifuge the plate at 500 × *g* at 4°C for 5 min.
  d. After centrifugation, visualize cell pellets then decant the supernatant by flicking the plate in one singular motion into a liquid waste container.
    i. Leave the plate momentarily inverted on a stack of low-particulate wipes to remove additional supernatant.
72. Extracellular stain – Trm Master Mix #1.
  a. Prepare Trm Master Mix #1 (Figure 5A). Resuspend samples in 50 μL of Trm Master Mix #1 (Table 3).
    ***Note:*** Researchers are encouraged to generate FMO/isotype controls for all fluorochromes. These should be prepared at this step (Figure 5B).
  b. Incubate samples for 30 min on ice, protected from light.
  c. Following incubation, add 150 μL of ice-cold FACS buffer to each well and centrifuge the plate at 500 × *g* at 4°C for 5 min.
  d. After centrifugation, visualize the cell pellets, then decant the supernatant.
73. Extracellular stain – Trm Master Mix #2.
  a. Prepare Trm Master Mix #2. Resuspend samples in 50 μL of Trm Master Mix #2 (Table 4).
    ***Note:*** Researchers are encouraged to generate FMO/isotype controls for all fluorochromes. These should be prepared at this step (Figure 5B).
  b. Incubate samples for 30 min on ice, protected from light.
  c. Following incubation, add 150 μL of ice-cold FACS buffer to each well and centrifuge the plate at 500 × *g* at 4°C for 5 min.
  d. After centrifugation, visualize the cell pellets, then decant the supernatant.
74. Intracellular stain – Trm Master Mix #3.
  a. Resuspend samples in 100 μL of Cytofix/Cytoperm and incubate samples for 20 min at 4°C, protected from light.
  b. Centrifuge the plate at 500 × *g* at 4°C for 5 min.
  c. After centrifugation, visualize the cell pellets, then decant the supernatant.
  d. Resuspend the samples in 200 μL of 1× Permwash solution and centrifuge the plate at 750 × *g* at 4°C for 5 min.
    **Pause point:** The fixed cell pellets can be resuspended in 200 μL of PBS and stored at 4°C for up to 24 h. Prior to proceeding with step 74e, centrifuge the plate at 750 × *g* at 4°C for 5 min, visualize the cell pellets, and decant the supernatant. Resuspend the samples in 200 μL of 1× Permwash solution, centrifuge the plate at 750 × *g* at 4°C for 5 min. After centrifugation, visualize the cell pellets, then decant the supernatant.
  e. Prepare Trm Master Mix #3. Resuspend samples in 50 μL of Trm Master Mix #3 and incubate samples for 30 min on ice, protected from light (Table 5).
  f. Following incubation, add 150 μL of 1× Permwash solution to each well and centrifuge the plate at 750 × *g* at 4°C for 5 min.
  g. After centrifugation, visualize the cell pellets, then decant the supernatant. Resuspend samples in 200 μL of FACs buffer. Samples are now ready for downstream flow cytometric analysis on flow cytometers such as the BD Biosciences LSR II, BD Biosciences Fortessa, BD Biosciences FACSymphony, Beckman Coulter Cytoflex, or Cytek Aurora.

### Immunological procedure #2: Flow cytometric analysis of trained immunity in alveolar macrophages

This section details the staining of BAL and lung mononuclear cells for indices of macrophage trained innate immunity. The staining and gating strategy also allows for downstream identification of multiple immune subsets including: (1) neutrophils, (2) monocytes, (3) alveolar macrophages, (4) interstitial macrophages, and (5) monocyte-derived macrophages (Hussell and Bell, 2014; Misharin et al., 2013).

The following protocol utilizes freshly isolated cells, however the same panel and gating strategy can also be applied to samples following *ex vivo* stimulation. Researchers should take into consideration that cell surface marker expression may be altered due to culture and stimulation conditions.

**Figure 6:**
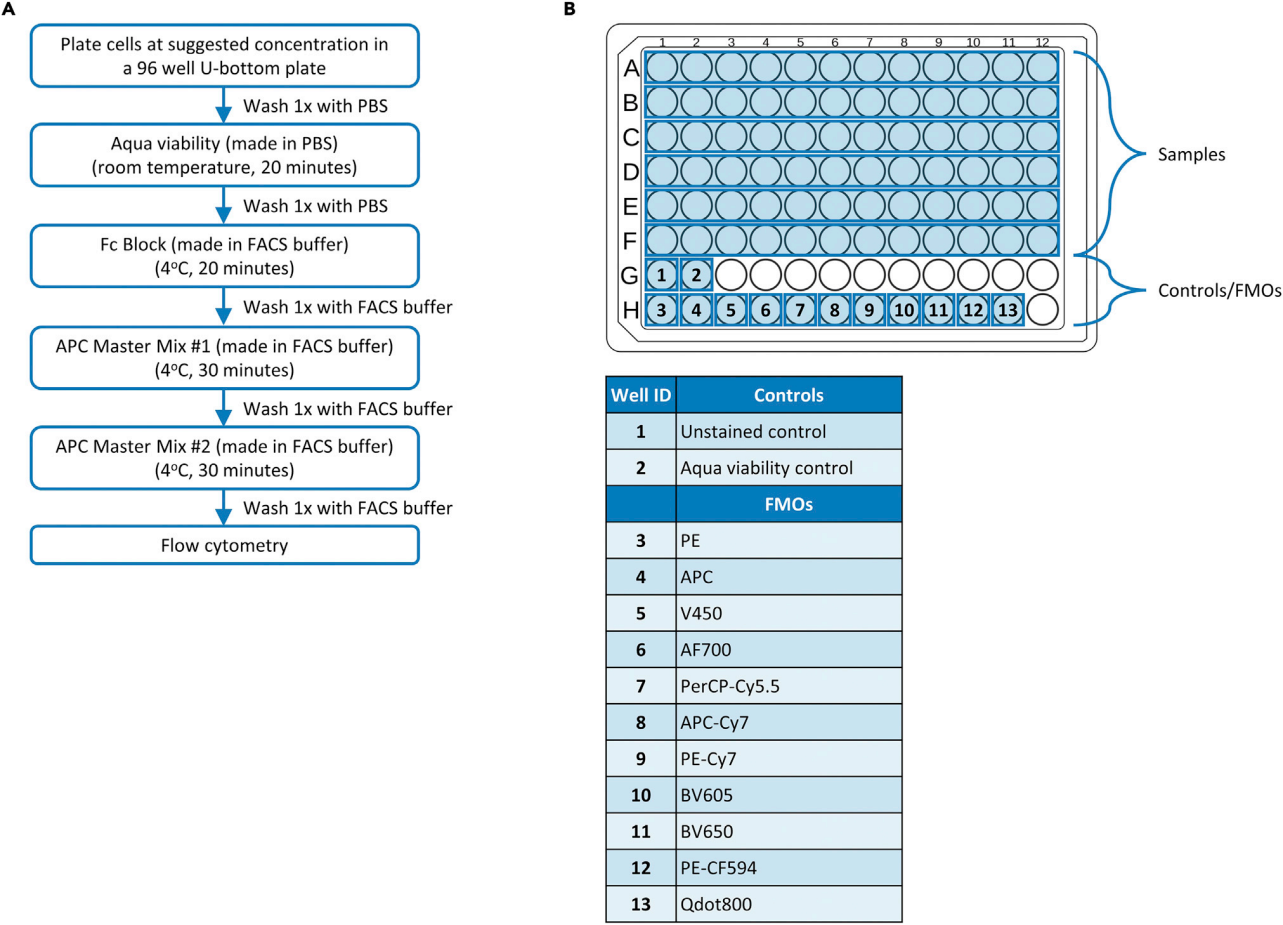
Staining strategy for identification of indices of macrophage trained innate immunity (A) Staining workflow. (B) Example plate layout.

The staining workflow for innate immune analysis is presented in Figure 6A. The antibody cocktail described herein is a suggestion for canonical pulmonary antigen-presenting cell markers using specific antibodies that we have experimentally proven to work. Researchers may need to adjust the panel as per experimental need.

**Timing: 3 h**

75. Mononuclear cell plating.
  a. Add volume/concentration-adjusted BAL and lung mononuclear cells to a 96 well U-bottom plate. To ensure enough events can be collected on the flow cytometer for sufficient data resolution, researchers are encouraged to: (1) Plate 5 × 10^5^–2 × 10^6^ lung mononuclear cells, and (2) 3–5 × 10^5^ BAL cells. Volume adjust cells to 200 μL.

***Note:*** Lung mononuclear cells may be utilized for FMO/isotype controls. Researchers are encouraged to prepare these for each colour to ensure proper quality control of positive population cut offs (Figure 6B).

76. Viability LIVE/DEAD staining protocol.
  a. Centrifuge the plate at 500 × *g* 4°C for 5 min.
  b. After centrifugation, visualize cell pellets then decant the supernatant by flicking the plate in one singular motion into a liquid waste container.
    i. Leave the plate momentarily inverted on a stack of low-particulate wipes to remove additional supernatant.
  c. Resuspend cell pellets with 200 μL of ice-cold PBS and centrifuge the plate at 500 × *g* at 4°C for 5 min.
    ***Note:*** The presence of serum/protein in the cell culture media may impact the resolution of amine reactive viability dyes. Researchers are encouraged to perform at least a single wash step with PBS prior to staining with amine reactive viability dyes as per manufacturer’s instruction.
  d. Prepare the viability stain working solution in PBS as per manufacturer’s instructions. Add 50 μL of the viability stain working solution to each well containing samples, except the unstained control (Table 6).
  e. Incubate the samples at 18°C–25°C for 20 min, protected from light.
    ***Note:*** For compensation, researchers may either prepare a cell color control stained with the viability dye (in this case, Aqua) or may utilize amine reactive beads (e.g., ArC amine reactive beads: Thermo Fisher Scientific cat# A10346).
  f. Following incubation, add 150 μL of ice-cold PBS to each well and centrifuge the plate at 500 × *g* at 4°C for 5 min.
  g. After centrifugation, visualize cell pellets then decant the supernatant by flicking the plate in one singular motion into a liquid waste container.
  h. Leave the plate momentarily inverted on a stack of low-particulate wipes to remove additional supernatant. Proceed immediately to the next staining step.
77. Fc Block staining protocol.
  a. Prepare the working dilution of Fc block and resuspend samples in 50 μL of the working dilution (Table 7).
  b. Incubate samples for 15 min on ice, protected from light.
  c. Following incubation, add 150 μL of ice-cold FACS buffer to each well and centrifuge the plate at 500 × *g* at 4°C for 5 min.
  d. After centrifugation, visualize cell pellets then decant the supernatant by flicking the plate in one singular motion into a liquid waste container.
  e. Leave the plate momentarily inverted on a stack of low-particulate wipes to remove additional supernatant.
78. Extracellular stain – Antigen-presenting cell (APC) Master Mix #1.
  a. Prepare APC Master Mix #1. Resuspend samples in 50 μL of APC Master Mix #1 (Table 8). If your antibody staining cocktail contains >2 brilliant dyes, researchers should consider using the BD Horizon Brilliant Stain Buffer (BD Biosciences Cat# 566349) during the staining as per manufacturer’s instructions.
    ***Note:*** Researchers are encouraged to generate FMO/isotype controls for all fluorochromes. These should be prepared at this step (Figure 6B).
  b. Incubate samples for 30 min on ice, protected from light.
  c. Following incubation, add 150 μL of ice-cold FACS buffer to each well and centrifuge the plate at 500 × *g* at 4°C for 5 min.
  d. After centrifugation, visualize the cell pellets, then decant the supernatant.
79. Extracellular stain – APC Master Mix #2.
  a. Prepare APC Master Mix #2. Resuspend samples in 50 μL of APC Master Mix #2 (Table 9).
    ***Note:*** Researchers are encouraged to generate FMO/isotype controls for all fluorochromes. These should be prepared at this step (Figure 6B).
  b. Incubate samples for 30 min on ice, protected from light.
  c. Following incubation, add 150 μL of ice-cold FACS buffer to each well and centrifuge the plate at 500 × *g* at 4°C for 5 min.
  d. After centrifugation, visualize the cell pellets, then decant the supernatant.
  e. Resuspend samples in 200 μL of FACs buffer. Samples are now ready for downstream flow cytometric analysis.

## Expected outcomes

### Gating strategy for phenotyping immune cells from the lungs and airways

The results herein are based on respiratory mucosal vaccination with a trivalent chimpanzee adenovirus-vectored COVID-19 vaccine (Afkhami et al., 2022). Following intranasal vaccination and pulmonary mononuclear cell isolation, appropriate flow cytometric staining techniques will allow for elucidation of resident memory T cells and assessment of indices of trained memory macrophages within both the lung parenchyma and airways.

**Figure 7:**
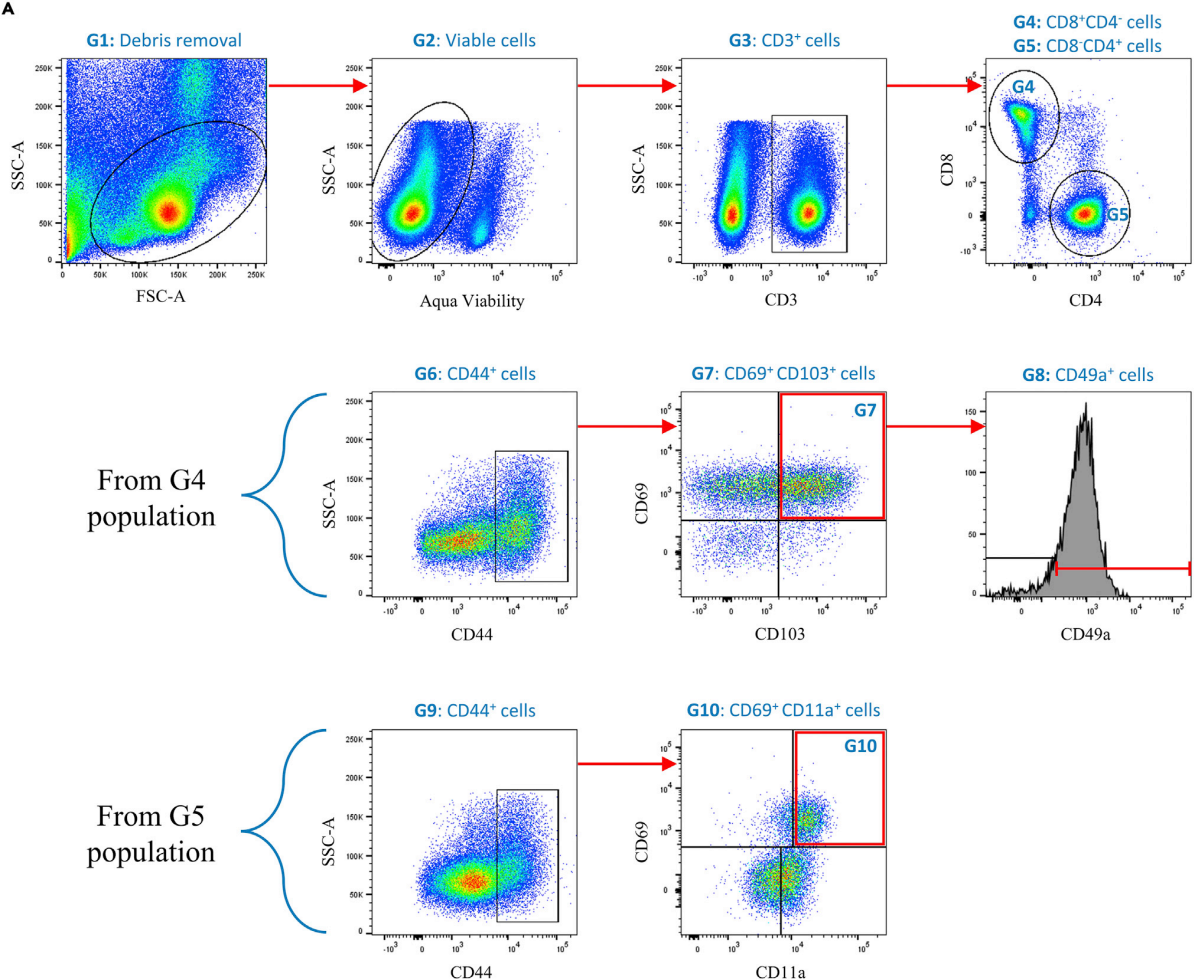
Gating strategy for identification of tissue resident memory T cells (A) Gating strategy for Trm T cells. Antigen experienced T cells are defined by their expression of CD44 (G6, G9). Following gating on CD44^+^ cells (G6), CD8^+^ Trm T cells are defined by their co-expression of CD69, CD103 (G7), and CD49a (G8). Following gating on CD44^+^ cells (G9), CD4^+^ Trm T cells are defined by their co-expression of CD69 and CD11a (G10).

The gating strategy presented in Figure 7 allows for definition of antigen-experienced T cells by their expression of CD44 (G6 and G9). Subsequently, CD8^+^ Trm T cells are defined by co-expression of CD69, CD103, and CD49a (G7 and subsequently G8), whereas CD4^+^ Trm T cells are defined by co-expression of CD69 and CD103 (G10). FMOs are frequently used as gating controls to establish the background signal levels caused by spillover from other channels. Gates where inclusion of FMOs will be of particular importance to aid in proper delineation of populations are for G6-G10.

**Figure 8:**
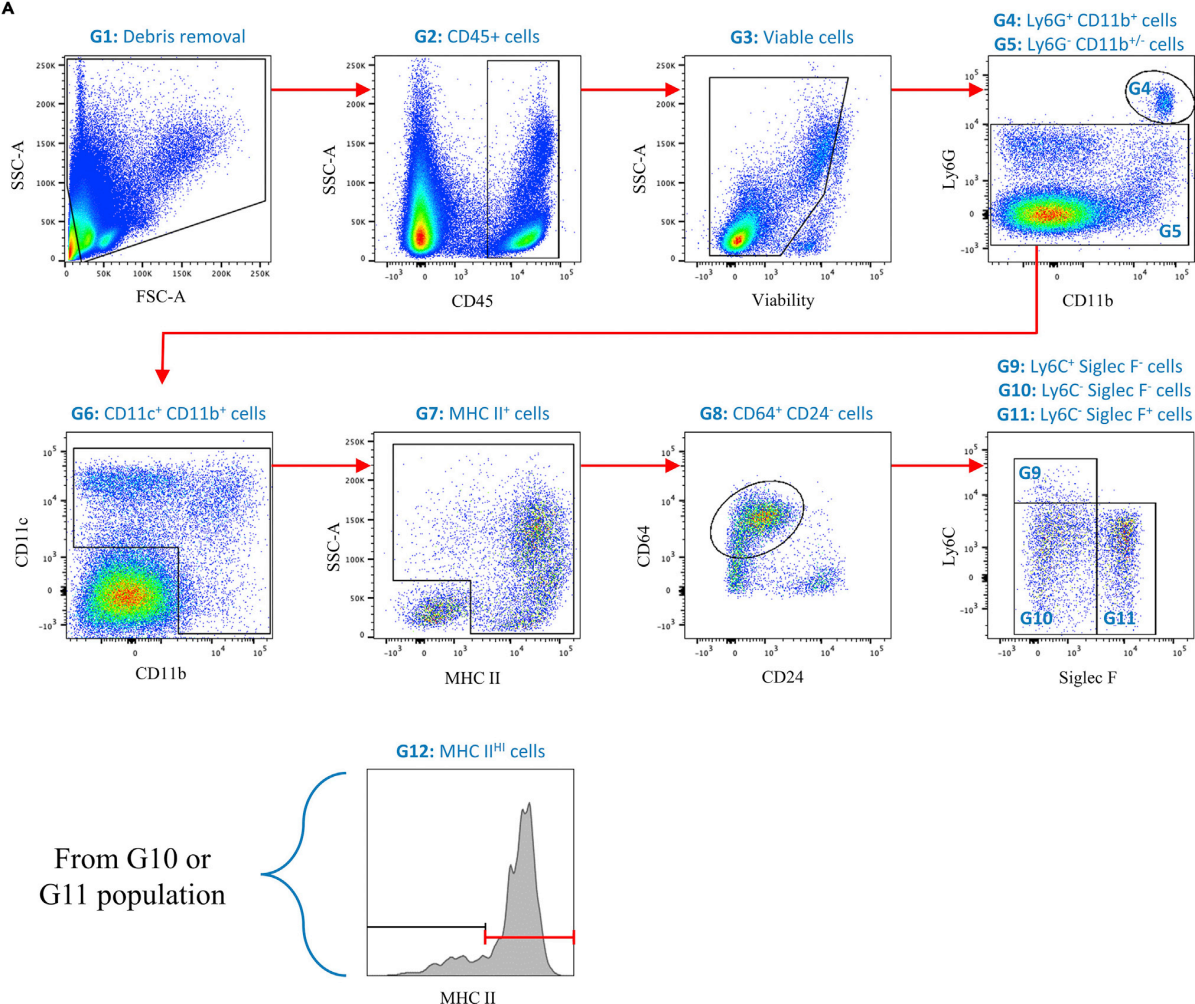
Gating strategy for trained airway macrophages (A) Gating strategy. Neutrophils are defined in this gating strategy by co-expression of Ly6G and CD11b (G4). Airway macrophages are defined by their expression of CD11c, CD11b, CD64, Ly6C and/or Siglec F (G9, G10, or G11). Cells in G9 are defined as monocyte-derived macrophages, G10 as interstitial macrophages, and G11 as alveolar macrophages. Increased MHC II expression is a canonical marker of trained immunity on airway macrophages (G12).

Additionally, a myriad of innate cell populations can be analyzed using the gating strategy from Figure 8. Gates where inclusion of FMOs will be of particular importance to aid in proper delineation of populations are for G4, and G9-11. This includes neutrophils (G4), monocyte-derived macrophages (G9), interstitial macrophages (G10) and alveolar macrophages (G11). A hallmark of pulmonary trained innate immunity on airway macrophages is stably increased expression of MHC II (G12). This can be quantified by measuring the mean fluorescent intensity (MFI) of MHC II on distinct airway macrophage populations such as alveolar macrophages or interstitial macrophages. In addition, acellular immune components such as mucosal immunoglobulins and cytokines can also be isolated, enabling further experimental readouts.

## Limitations

The efficiency and reproducibility of the intranasal vaccination in mice with respiratory dysfunction may be adversely impacted. Researchers may notice the mice failing to inhale the liquid or intermittently having the liquid bubble out of their nose. Adjustments in (1) anesthesia level, (2) instillation volume, and (3) rate of instillation may be required. Insults which adversely impact lung integrity may impair BAL recovery. Leaks during the BAL procedure may be addressed by (1) lowering the volume of PBS used for BAL, (2) reducing the number of lavages performed, and (3) limiting how much the lungs are massaged.

## Troubleshooting

### Problem 1

Trachea slips off the BAL needle (step 35).

### Potential solution

This is often caused by the suture not being sufficiently tightened around the BAL needle. This can be minimized by ensuring the suture is hand-tightened.

### Problem 2

Uneven lung inflation following PBS instillation (step 36).

### Potential solution

The depth and angle of the BAL needle in the trachea may need to be adjusted. In this case, aspirate the instilled PBS, readjust the BAL needle and tighten the suture, then proceed with steps 35–37.

### Problem 3

PBS leaking from the trachea during BAL (step 36).

### Potential solution

Re-insert the BAL needle past the leak. Additional sutures may be required.

### Problem 4

PBS leaking from the lungs during BAL (step 36).

### Potential solution

Reducing the frequency and intensity of which the lungs are massaged during the BAL procedure.

### Problem 5

Cytokines or immunoglobulins are not detectable from the conventional BAL (step 48).

### Potential solution

Consider concentrating the conventional BAL fluid using Pierce^TM^ protein concentrators PES (Thermo Fisher Scientific Cat# 88504) or equivalent.

### Problem 6

Macrophage populations autofluoresce in the 350–550 nm range, particularly in the FITC channel and can spill into overlapping channels such as Aqua and PE (steps 75–79).

### Potential solution

Carefully consider where to draw the gates during analysis, as well as using certain viability dyes such as Aqua, or antibodies conjugated to fluorochromes such PE, or FITC.

## Resource availability

### Lead contact

Further information and requests for resources and reagents should be directed to and will be fulfilled by the lead contacts, Michael D’Agostino (dagostim@mcmaster.ca) and Sam Afkhami (afkhams@mcmaster.ca).

### Materials availability

This study did not generate new unique reagents.

## Data Availability

All data supporting the findings of this study are available within the paper and are available from the lead contact or correspondence authors upon request. This study did not generate/analyze original datasets or code.
